# Kullback–Leibler Divergence Based Probabilistic Approach for Device-Free Localization Using Channel State Information

**DOI:** 10.3390/s19214783

**Published:** 2019-11-03

**Authors:** Ruofei Gao, Jie Zhang, Wendong Xiao, Yanjiao Li

**Affiliations:** 1 School of Automation & Electrical Engineering, University of Science and Technology Beijing, Beijing 100083, China; g20178585@xs.ustb.edu.cn; 2 Beijing Engineering Research Center of Industrial Spectrum Imaging, Beijing 100083, China; 3 School of Electronics Engineering and Computer Science, Peking University, Beijing 100871, China; zhangjie.saee@pku.edu.cn; 4 School of Information and Electronics, Beijing Institute of Technology, Beijing 100081, China; yanjiaoli@bit.edu.cn

**Keywords:** device-free localization, channel state information, multivariate Gaussian distribution, Kullback–Leibler divergence, amplitudes, phases

## Abstract

Recently, people have become more and more interested in wireless sensing applications, among which indoor localization is one of the most attractive. Generally, indoor localization can be classified as device-based and device-free localization (DFL). The former requires a target to carry certain devices or sensors to assist the localization process, whereas the latter has no such requirement, which merely requires the wireless network to be deployed around the environment to sense the target, rendering it much more challenging. Channel State Information (CSI)—a kind of information collected in the physical layer—is composed of multiple subcarriers, boasting highly fined granularity, which has gradually become a focus of indoor localization applications. In this paper, we propose an approach to performing DFL tasks by exploiting the uncertainty of CSI. We respectively utilize the CSI amplitudes and phases of multiple communication links to construct fingerprints, each of which is a set of multivariate Gaussian distributions that reflect the uncertainty information of CSI. Additionally, we propose a kind of combined fingerprints to simultaneously utilize the CSI amplitudes and phases, hoping to improve localization accuracy. Then, we adopt a Kullback–Leibler divergence (KL-divergence) based kernel function to calculate the probabilities that a testing fingerprint belongs to all the reference locations. Next, to localize the target, we utilize the computed probabilities as weights to average the reference locations. Experimental results show that the proposed approach, whatever type of fingerprints is used, outperforms the existing Pilot and Nuzzer systems in two typical indoor environments. We conduct extensive experiments to explore the effects of different parameters on localization performance, and the results demonstrate the efficiency of the proposed approach.

## 1. Introduction

Recent years have seen the rapid development of wireless network technology, and people are demanding more effective and more precise services. Indoor localization is definitely one of them. Compared to outdoor localization, which mostly resorts to Global Positioning System (GPS) to implement an application, indoor localization, because of the environmental factors like multipath effects, shadowing, and fading, is a much more challenging task. Researchers have proposed different approaches to performing an indoor localization task, aiming to achieve higher accuracy. Most of the existing approaches are device-based, which have a major drawback that the target needs to equip itself with a certain device in advance. However, in some cases, it is unreasonable to require the subject to carry any devices. For example, in an intrusion detection and localization application, intruders will not equip themselves with devices to communicate with the central system, making the device-based approaches inapplicable.

To overcome the problems existing in device-based localization, Youssef et al. [1] introduced the concept of device-free localization (DFL), which eliminates the need to have any device attached to a target and merely requires the wireless network to be deployed around the environment to sense the target. Since then, DFL has gradually become the focus of indoor localization, and a lot of approaches have been presented. Among them, fingerprinting-based approaches are one of the most popular kinds. A fingerprinting-based approach consists of an offline phase and an online phase. The offline phase focuses on constructing a radio map that stores the fingerprints of reference locations, whereas the online phase aims to estimate a target’s location by comparing the newly collected measurements with the radio map. For example, Seifieldin et al. [2] presented Nuzzer, which utilizes Received Signal Strength Indication (RSSI) from different data streams to construct the radio map as histograms. Xu et al. [3] determined a target’s location through classification by incorporating a probability-based approach and discriminant analysis. However, these approaches are based on RSSI, which is easy to retrieve with low hardware cost but values of which are susceptible to the multipath effect. RSSI has strong innate variability, causing its value to fluctuate over time. Furthermore, RSSI is rather coarse-grained, because it merely uses an integer value to represent the quality of a communication link. Channel State Information (CSI)—a kind of information extracted from the physical layer—is based on Orthogonal Frequency Division Multiplexing (OFDM), which transmits data through different subcarriers. Therefore, CSI can characterize the quality of a communication link with multiple values, which has more fined granularity than RSSI. Through the use of CSITOOL [4], CSI can be easily retrieved from an Intel 5300 wireless card. Recently, there have been some applications on DFL adopting CSI as the basic measurements. For example, Xiao et al. [5] proposed a system to construct the radio map using the stability of CSI, and they adopt a kernel-density based approach to determine the location of a target. Zhou et al. [6] utilized Support Vector Machine (SVM) to establish the dependency relationship between the CSI amplitudes and the target’s location. Most of the existing CSI-based DFL approaches either merely use the CSI amplitudes or phases to construct fingerprints, or only adopt one antenna to collect measurements, potentially discarding a large portion of useful information. Furthermore, at the online phase, they tend to compare one sample each time with the radio map to localize the target, neglecting the information among successive samples within a period.

In this study, we present a novel approach that not only incorporates the CSI measurements of multiple communication links but also exploits the uncertainty information among successive samples, which is embodied by a probability distribution, to implement indoor localization. The proposed approach can utilize the CSI amplitudes or phases to localize a target with Kullback–Leibler divergence (KL-divergence). Also, it can simultaneously utilize the information of the CSI amplitudes and phases.

Specifically, we perform four statistical analyses to explore the characteristics of CSI. We then introduce the proposed approach, which is composed of a communal processing module, an offline fingerprint generation module, and an online KL-divergence based localization module. The part of the work of the processing module is to sanitize the raw CSI phases with a linear transformation to make sure they are usable. Furthermore, we model the CSI amplitudes and sanitized phases of all the subcarriers within a communication link as multivariate Gaussian distributions. Therefore, we need to estimate the mean vectors and covariance matrices of them. The processing module plays that role and also handles the problem with non-invertible situations occurring in the parameter estimation process. The offline fingerprint generation module receives the estimated parameters at the offline phase from the processing module and then records them as the reference fingerprints to form a radio map. The online KL-divergence based localization module is aimed to compare the fingerprints of a target estimated at the online phase with the radio map to localize the target by utilizing KL-divergence. Moreover, the proposed approach can process three different types of fingerprints, i.e., the amplitude fingerprints, the phase fingerprints, and the combined fingerprints, which are the combination of the amplitude fingerprints and the phase fingerprints.

We conduct extensive experiments in two typical indoor environments, a corridor and laboratory room, to demonstrate the effectiveness of the proposed approach. The results show that the proposed approach, using whatever type of fingerprints, achieves better performance than CSI-based Pilot and RSSI-based Nuzzer. In addition, we also explore the sensitivity of different parameters to the localization performance.

The rest of this paper is organized as follows. Section 2 presents some reviews about existing works on indoor localization. Section 3 articulates relevant preliminaries of this study. We present in Section 4 some characteristics of the CSI amplitudes and phases based on statistical tests. Section 5 introduces the structure of the proposed approach. In Section 6, we show the results of the proposed approach and the effects of different parameters on localization accuracy. Finally, we conclude the paper in Section 7.

## 2. Related Works

Indoor localization applications can be broadly classified as device-based and device-free kinds, depending on their requirements, i.e., whether or not the target needs to equip itself with other assistant devices.

### 2.1. Device-Based Indoor Localization

Device-based indoor localization applications use the signal transmitted directly from the device carried by the target to perform a localization task. For example, Want et al. [7] used active badges to localize a target. Aparicio et al. [8] utilized Bluetooth to estimate a target’s location. Ni et al. [9] deployed RFID tags around the monitoring area. Hazas et al. [10] addressed the localization problem by incorporating ultrasound. However, these approaches need either special hardware or dense deployment of devices, which limits their large-scale use. Wi-Fi, which is extremely pervasive nowadays and has low hardware requirements, is an alternative to these techniques. RADAR [11], a Wireless Local Area Network (WLAN) based localization system, which can be implemented with Wi-Fi devices easily, extracts RSSI from Wi-Fi devices and constructs a radio map at the offline phase, and then compares the measurements collected at the online phase with the radio map to estimate the location. Horus [12] incorporates a probabilistic approach when estimating the location at the online phase, finally achieving much better performance than RADAR.

Except RSSI, CSI is also a kind of information that we can retrieve from Wi-Fi, and there are also some works focusing on it. PILA [13], using the collected CSI measurements to estimate the Angle of Arrival (AoA) information, handles the localization task by solving the defined objective function with the AoA information and RSSI. FILA [14], aggregating the CSI of different subcarriers to alleviate the negative effects incurring by the environmental factors, proposed an improved propagation model to enhance the localization accuracy. FIFS [15] collects the CSI information from multiple antennas and sums them up, further averaging the aggregated CSI information of all subcarriers to represent each unique location. CSI-MIMO [16] explores the CSI amplitudes and phases in their work by considering the difference of the amplitudes or phases between adjacent subcarriers as the fingerprints. Zheng et al. [17] proposed a fingerprinting-based approach that utilizes the signatures obtained from the CSI to enhance localization performance. Want et al. [18] proposed an approach that utilizes a random forest to train the data collected at the offline phase and predict the target’s location at the online phase. Furthermore, deep learning-based approaches have also been incorporated to improve accuracy. For example, DeepFi [19] and PhaseFi [20] both use deep learning algorithms to generate fingerprints and then adopt a probabilistic method to localize the target, reporting better performance than FIFS. ConFi [21] constructs its radio map by considering the fingerprints as images and then exploits a Convolutional Neural Network (CNN) to localize the target, yielding better results than DeepFi in its experiments.

### 2.2. Device-Free Indoor Localization

As we have mentioned, device-based indoor localization seems to be infeasible when applied to certain scenarios, whereas the device-free kind can adapt itself to them.

Youssef et al. [1] first introduced the concept and architecture of DFL, and they also demonstrated its feasibility. Since then, DFL studies have surged up. DFL can be broadly classified as model-based and fingerprinting-based approaches. Model-based approaches generally aim to find the relationship between the signal and the target’s location and render it as a mathematical problem. For example, Wilson et al. [22] proposed a novel approach called Radio Tomographic Imaging (RTI), inspired from the concept of tomographic imaging, to constructing the images of a target’s location based on RSSI by using a linear model. Based on RTI, some improved approaches were also proposed [23,24]. WiTrack [25] exploits the signal reflected off the target and incorporates a geometric approach to localize the target. Dynamic-Music [26] proposed a novel approach to detect the reflected signal off the human body using the CSI phase measurements and further compute the Angle of Arrival (AoA) and Time of Arrival (ToA) to estimate the location information. IndoTrack [27] derives Doppler velocity from CSI measurements and estimates the target’s location and velocity information, which reported a submeter level localization accuracy in their experiments. Widar [28] exploits CSI to localize a target and simultaneously present the target’s velocity estimate, achieving a localization accuracy of below one meter. Widar2.0 [29] enhances the Widar system by using only one Wi-Fi link, the results also showing a decimeter-level accuracy. Xiao et al. [30] presented an approach to identifying the affected communication links and localizing a target from a view of optimization. Though the model-based approaches usually display rather excellent performance, they struggle to handle the scenarios with cluttered environmental settings, which limits their real use.

Fingerprinting-based DFL techniques aim to record the impacts on the signal caused by a target standing at different locations and store these impacts into a radio map. When localizing a target, they usually compare the online wireless measurements with the radio map and then incorporate a deterministic or probabilistic approach to estimate the location information. Nuzzer utilizes histograms to characterize the distributions of RSSI when a target stands at different locations and uses a probabilistic approach to compute several most similar fingerprints in the radio map to estimate the target’s position. PC-DfP proposed a classification-based localization approach that exploits discriminant analysis to enhance accuracy, finally obtaining excellent performance in noisy environments. Pilot, adopting CSI as its basic measurements, detects if there is a target in the monitoring area and uses Kernel Density Estimation (KDE) to estimate the distribution of the correlation between the abnormality patterns and normality profile. Pilot achieved better performance than a Nuzzer-like approach in its experiments. Moreover, some researchers also incorporate machine learning and deep learning algorithms to implement a DFL application. Zhang et al. [31] presented an approach that combines parameterized geometrical feature extraction (PGFE) with Extreme Learning Machine (ELM) to perform a localization task. Zhang et al. [32] proposed an ELM algorithm incorporated with a residual compensation strategy and demonstrated its efficiency in an RSSI-based DFL application. Additionally, probability-based machine learning algorithms, such as multilayer probability ELM (MP-ELM) [33], are also proposed to implement a DFL application. Gao et al. [34] utilized an ELM Ensemble together with Principal Component Analysis (PCA) to implement a DFL application. Gao et al. [35] used a deep learning approach in order to learn features from CSI radio signals and adopted softmax-regression to predict the location information.

## 3. Preliminaries

### 3.1. Channel State Information

CSI, complying with the standards of OFDM, consists of multiple subcarriers, each of which has a channel gain composed of amplitude and phase. Furthermore, the amplitudes and phases of different subcarriers are generally different from each other, extraordinarily boosting the information contained in CSI. CSI has the ability to characterize the effects of multipath effect, fading, and transmission delay. In other words, CSI reveals how a signal is transmitted between transceivers. CSI can be easily retrieved using CSITOOL, which merely requires a device installed with an intel 5300 Wi-Fi wireless card.

For an OFDM system, we use X and Y to represent the signals to be transmitted and received respectively. We further represent its model in the same form as in [15,16]:

(1) Y=ϕX+ε,

where ϕ is the channel matrix. The term ε denotes the additive white Gaussian noise. Further neglecting the term ε, we can derive an estimate of the channel matrix according to Equation (1). The channel matrix we retrieve by using CSITOOL has a dimension of *M_t_* × *M_r_* × *M_s_*, where $M_{t}$ is the number of antennas for signal transmission, *M_r_* denotes the number of antennas for signal reception, and $M_{s}$ represents the number of subcarriers within a communication link. Moreover, CSITOOL allows us to retrieve 30 subcarriers within a communication link. Therefore, in this paper, $M_{s}$ is equal to 30. Furthermore, for an arbitrary element *φ* in ϕ, we denote it as:

(2) $$\varphi =\left|\varphi \right|e^{j∠\varphi },$$

where |φ| is the amplitude and ∠φ the phase of a subcarrier.

### 3.2. Preprocessing

For the CSI raw phases, because of their innate randomness, they display no certain pattern that we can capture. According to [36], we adopt a phase sanitization algorithm to solve this problem, working well and efficient, which uses a linear transformation with merely two parameters to transform the raw phases into usable phases. Next, we will briefly introduce the algorithm. For subcarrier *j*, its raw phases can be represented as follows:

(3) $${\hat{P}}_{i}=P_{i}-2\pi \frac{c_{i}}{N}\Delta \psi +\omega +Z,$$

where $P_{i}$ is the ground truth of the phase of subcarrier i, Δψ is the time lag produced at the receiver, ω is the initial phase offset, and *Z* is the noise term. $c_{i}$ is the subcarrier index and N is the FFT size. The randomness is mainly caused by ω and Δψ, which are two unknowns. We first define the following variables, with Z neglected (assuming that there are k subcarriers within a communication link):

(4) $$\theta =\frac{{\hat{P}}_{k}-{\hat{P}}_{1}}{c_{k}-c_{1}},$$

(5) $$\lambda =\frac{1}{k}\overset{k}{\underset{j=1}{\sum }}{\hat{P}}_{j}.$$

Then, we can obtain the sanitized phase by subtracting $c_{i}\theta +\lambda$ from the raw phase ${\hat{P}}_{i}$, written as:

(6) $${\tilde{P}}_{i}={\hat{P}}_{i}-c_{i}\theta -\lambda .$$

In doing so, we can mitigate or eliminate the effects of the unknowns, thus producing usable phase information.

**Figure 1:**
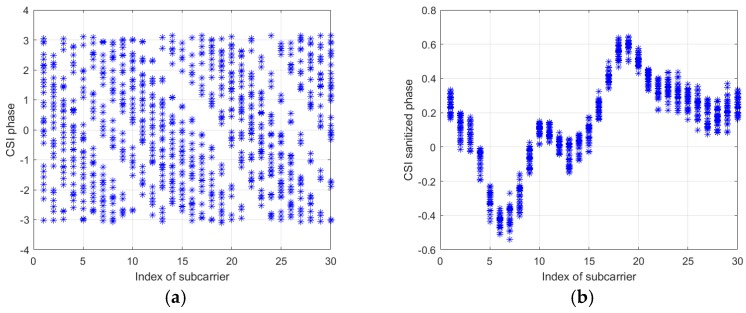
Comparison of (**a**) the Channel State Information (CSI) raw phases with (**b**) sanitized phases.

Figure 1 shows the contrast between the raw phases and the sanitized phases of 30 subcarriers, where we can see that the raw phases after unwrapping lie in all feasible region between [−π,π] whereas the sanitized phases lie in a more concentrated area.

Furthermore, it is easy to know that, noises neglected, the sanitized phases of the first subcarrier are the same as those of the last subcarrier, within a communication link. They share the same form, written as:

(7) $${\tilde{P}}_{k}={\tilde{P}}_{1}=\frac{c_{k}{\hat{P}}_{1}-c_{1}{\hat{P}}_{k}}{c_{k}-c_{1}}-\lambda .$$

Therefore, in Section 6 of this paper, to reduce redundancy within a communication link, we retain the sanitized phases of the first subcarrier, while neglecting those of the last.

## 4. Statistical Analyses

In this section, we analyze the characteristics of the CSI amplitudes and sanitized phases using several statistical tests, which we can use to support the proposed approach.

### 4.1. Analysis 1

As we have presented above, the CSI sanitized phases are more concentrated, but we can see that they still fluctuate, meaning there is uncertainty over consecutive samples. Furthermore, we notice there are certain patterns over the uncertainty, which can be characterized by probability distributions. Therefore, in this part, we try to figure out what distribution the CSI sanitized phases approximately exhibit when no target or a target is standing still in a monitoring area, and we use statistical experiments to demonstrate that the Gaussian distribution is a possible candidate.

To test if the sanitized phases of a subcarrier can be modeled as a Gaussian distribution, we perform a Shapiro–Wilk test in an indoor environment. The Shapiro-Wilk test is a kind of normality test, which presents a hypothesis that the data for testing obey a Gaussian distribution, and there is a value p denoting whether we should reject the hypothesis. Generally, if p is greater than a threshold, we have no reason to reject the hypothesis. In this study, we hold that if p is greater than 0.05, we cannot reject the hypothesis, so in this case, for simplicity, we are forced to accept the hypothesis. We present a variable to indicate whether or not to reject the hypothesis, written as

(8) $$S\left(p\right)=\{\begin{array}{cc} 0, & if\;p>0.05\\ 1, & others \end{array},$$

where the value of S(p) is either 0 or 1. 0 denotes that the hypothesis is not rejected and 1 means it is rejected.

We first perform the normality test when the monitoring area is empty, meaning that no target is present in the area. We define a rejection ratio $r_{e}$ to indicate the proportion of subcarriers that are rejected, written as:

(9) $$r_{e}=\frac{\sum_{i=1}^{k}S\left(p_{i}\right)}{k}$$

where $p_{i}$ is the p value of the subcarrier *i*, k is the total number of subcarriers in a communication link. We collect 50 consecutive samples at five different moments respectively and adopted the average of their rejection ratios as the final result, which is shown in Table 1. We can see that the value of $r_{e}$ is 0.0556, meaning that when there is no target in the monitoring area, the sanitized phases of over 94% of the subcarriers are not rejected.

Next, we conducted experiments when the target was present in the monitoring area. We modify the original rejection ratio as follows to measure the overall level of how many subcarriers are rejected in this area:

(10) $$r_{e}^{\prime }=\frac{\sum_{j=1}^{L_{N}}r_{e}{}_{j}}{L_{N}},$$

where $r_{e}{}_{j}$ is the rejection ratio of the location j, $L_{N}$ is the total number of locations. Also, we tested at five different moments, and adopted the average value of them for verification. According to Table 1, we can see that the value of $r_{e}^{\prime }$ is 0.1235, indicating that over 87% of the subcarriers are not rejected.

By comparing the results tested in the two conditions, it is easy to see that when the monitoring area was empty of the target, the rejection ratio is lower than that when the target stood in the area. This may be caused by the combined effects of the environment and the target. There are generally noises in the environments, which will cause unexpected fluctuations to the signal. Also, the target, which is the human body in this study, will further introduce noises to the signal. Therefore, the combined effects of them may raise the rejection ratio to a higher level.

**Figure 2:**
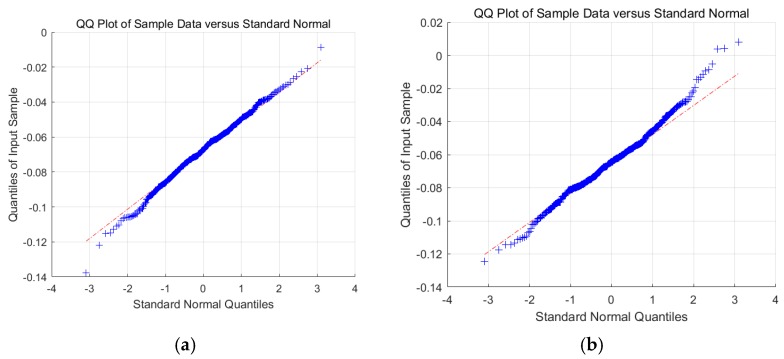
Quantile–quantile (QQ)-plot of a subcarrier (**a**) when the monitoring area is empty and (**b**) when the target stands at a location in the monitoring area.

We only use 50 consecutive samples to perform Shapiro–Wilk test, and for the situations with more consecutive samples, we use quantile–quantile plot (QQ-plot) to perform the test. We only show the results of a subcarrier because the results of different subcarriers are similar to one another. According to Figure 2, we can see that when the area is empty of the target, almost all the points follow along the straight line, with few of them far from the line. This phenomenon reveals that we can model the sanitized phases of this subcarrier as a Gaussian variable with great confidence. However, when the target is present in the area, the points at the upper right part start to tip away, but most of the points still stick to the straight line. In this situation, when having high acceptability, we can still consider the sanitized phase of this subcarrier as an approximately Gaussian variable.

According to the results, we consider that the CSI sanitized phase of a subcarrier can be modeled as an approximately Gaussian variable when there is no target or a target standing still in a monitoring area.

### 4.2. Analysis 2

**Figure 3:**
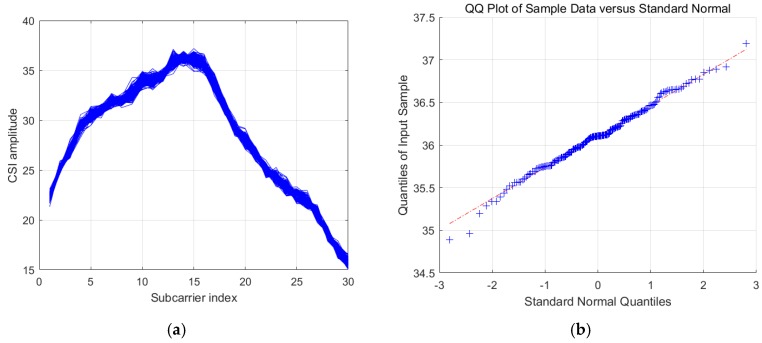
(**a**) The situation where CSI amplitudes form one cluster; (**b**) the QQ-plot of a subcarrier whose amplitudes exhibit an approximately Gaussian distribution.

**Figure 4:**
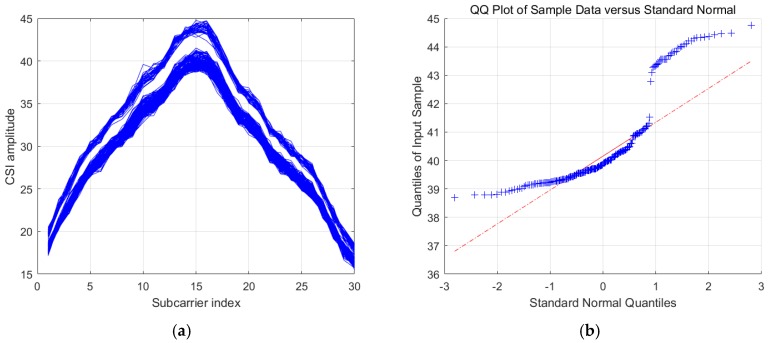
(**a**) The situation where CSI amplitudes form two clusters; (**b**) the QQ-plot of a subcarrier whose amplitudes exhibit a non-Gaussian distribution.

In this part, we explore the distribution of the CSI amplitudes. In comparison to the CSI sanitized phases, CSI amplitudes do not have stable uncertainty patterns we can capture. Sometimes, they exhibit an approximately Gaussian distribution, whereas other times they do not. According to Figure 3a, we can see that the CSI amplitudes from a sequence of consecutive samples are considerably close to one another, finally forming a cluster. In Figure 3b, we show the QQ-plot of the CSI amplitudes of the 15th subcarrier, where we can see that the CSI amplitudes of this subcarrier can be approximately modeled as a Gaussian distribution. However, as shown in Figure 4a, we can see a situation where the CSI amplitudes display another form of distribution with two clusters. Furthermore, Figure 4b shows the QQ-plot of the CSI amplitudes of the 15th subcarrier, where we can conclude that the CSI amplitudes of this subcarrier cannot be modeled as a Gaussian distribution.

In this study, to better utilize the information of the CSI amplitudes’ uncertainty without too much effort, we also model the CSI amplitudes of a subcarrier as a Gaussian distribution, which will simplify the consequent localization implementation.

### 4.3. Analysis 3

In this part, we conducted several experiments to explore the effects of a target’s location on the CSI amplitudes and sanitized phases of a communication link. Furthermore, to better illustrate these effects, we use the mean vector and the covariance matrix of the CSI amplitudes or phases of all the subcarriers from a communication link to show the results.

**Figure 5:**
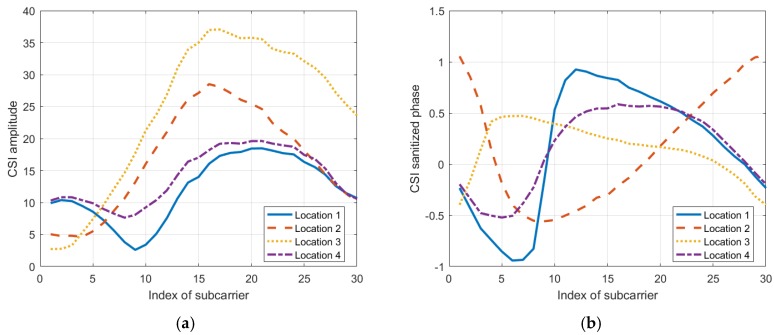
The mean vectors of a communication link tested at 4 locations for (**a**) the CSI amplitude and (**b**) the CSI sanitized phase.

**Figure 6:**
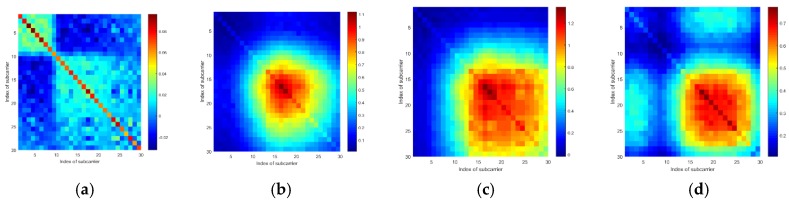
The CSI amplitude’s covariance matrices of a communication link tested at 4 different locations: (**a**) Location 1, (**b**) Location 2, (**c**) Location 3, and (**d**) Location 4.

**Figure 7:**
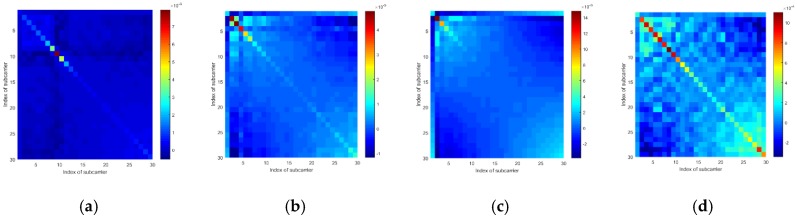
The CSI sanitized phase’s covariance matrices of a communication link tested at 4 different locations: (**a**) Location 1, (**b**) Location 2, (**c**) Location 3, and (**d**) Location 4.

To examine if the target standing at different locations will lead the mean vectors and the covariance matrices to exhibit different patterns, we tested at four locations. Additionally, to eliminate the effects of the human body’s motions, we used a metal box to represent the target. According to Figure 5, we can see that when the target locates at different positions, the mean vectors of the CSI amplitudes and the sanitized phases are generally different from one another. Also, according to Figure 6 and Figure 7, the covariance matrices at different locations display various patterns.

According to the results, we consider that the response of the CSI amplitudes and sanitized phases are affected by where a target stands, and therefore, the mean vectors and covariance matrices can be used to discriminate among locations.

### 4.4. Analysis 4

Because of the Multiple-Input Multiple-Output (MIMO) technology, we can transmit signals using multiple communication links, thus making it possible for us to exploit this technique to boost information. In this part, we look into the response of different communication links to the same environment context by exploring their mean vectors and covariance matrices.

**Figure 8:**
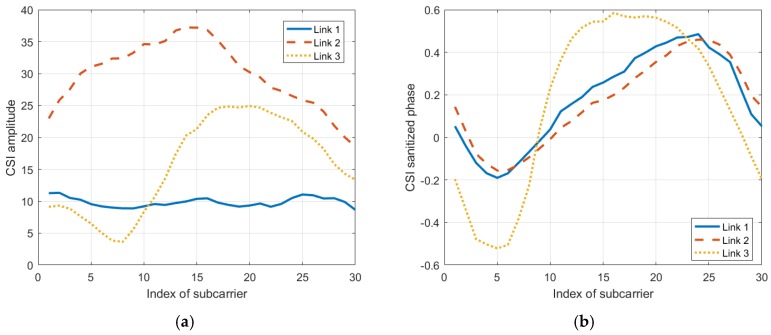
The mean vectors of (**a**) the CSI amplitude and (**b**) the CSI sanitized phase from 3 different communication links.

**Figure 9:**
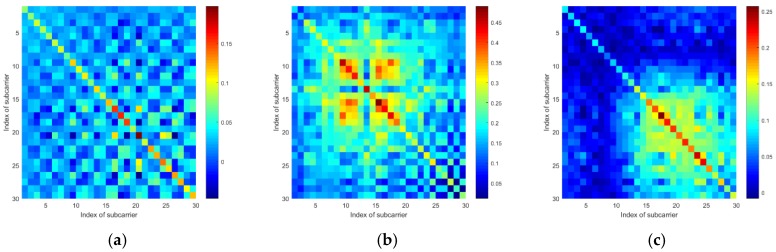
The CSI amplitude’s covariance matrices of 3 different communication links: (**a**) Link 1, (**b**) Link 2, and (**c**) Link 3.

**Figure 10:**
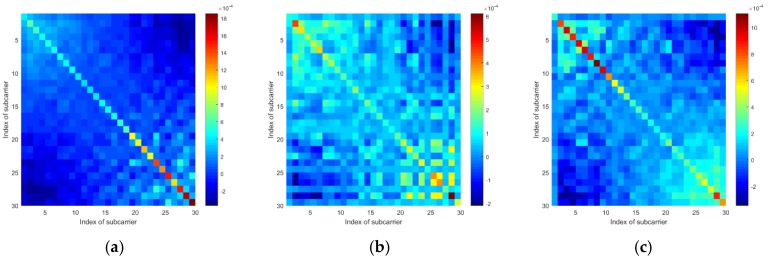
The CSI sanitized phase’s covariance matrices of 3 different communication links: (**a**) Link 1, (**b**) Link 2, and (**c**) Link 3.

According to Figure 8, the mean vectors of the CSI amplitudes are rather different from one another, so are the mean vectors of the CSI sanitized phases. Moreover, as shown in Figure 9 and Figure 10, we can also see great differences in covariance matrices of different communication links for either the CSI amplitudes or the CSI sanitized phases.

According to these results, we consider that it is reasonable to incorporate multiple communication links to boost the information in the radio map, which may further improve localization accuracy.

## 5. System Design

### 5.1. Overall Architecture

**Figure 11:**
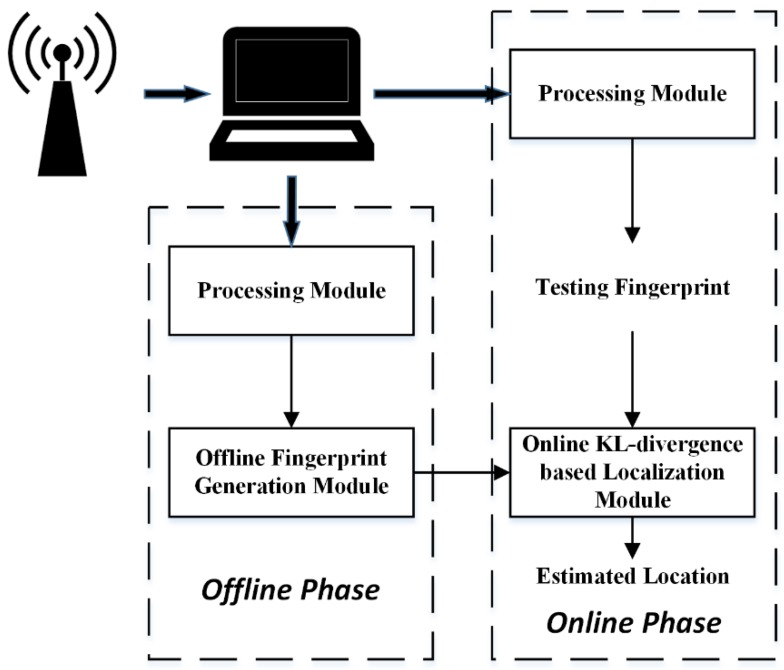
The architecture of the proposed approach.

Figure 11 shows the overall architecture of the proposed approach, which has three major modules, i.e., the processing module, the offline fingerprint generation module, and the online KL-divergence based localization module. Next, we will present the details of each module.

### 5.2. Processing Module

To better exploit the uncertainty information and according to the statistical analyses presented in Section 4, we consider that the CSI amplitudes and sanitized phases of all the subcarriers within a communication link can be modeled as a multivariate Gaussian distribution, written as

(11) $$f\left(v\right)=\frac{1}{\sqrt{{\left(2\pi \right)}^{k}}\left|\Sigma \right|}e^{\left(-\frac{1}{2}{\left(v-\mu \right)}^{T}\Sigma^{-1}\left(v-\mu \right)\right)},$$

where μ is the expectation vector of $v={\left[v_{1},v_{2},\ldots ,v_{k}\right]}^{T}$, $v_{i}$ is either the CSI amplitude or the sanitized phase of the subcarrier i, and Σ is the covariance matrix. The main goal of the processing module, a communal module used in the offline phase as well as the online phase, is to fit these multivariate Gaussian distributions.

First of all, the processing module will divide raw CSI measurements into amplitudes and phases and perform different operations depending on what type of fingerprints we are about to use. For instance, if the CSI amplitudes are adopted to construct the fingerprints, the processing module will directly perform a fitting operation on them. When the CSI phases are used for the construction of the fingerprints, the processing module will first sanitize them using the aforementioned linear transformation and then implement the fitting operation. Furthermore, if both are used, the above two processes will be carried on simultaneously.

Specifically, during the fitting operation, we aim to estimate the parameters of the distribution represented in Equation (11). To begin with, we transform it to the log-scale as follows:

(12) $$L\left(v\right)=-\frac{k}{2}\ln\left(2\pi \right)-\frac{1}{2}\ln\left(\left|\Sigma \right|\right)-\frac{1}{2}{\left(v-\mu \right)}^{T}\Sigma^{-1}\left(v-\mu \right).$$

Then, for a sequence of data composed of m samples $D=\left\{D_{1},\ldots ,D_{m}\right\}$, assuming these samples are independent identically distributed, we can easily obtain the estimated parameters $\hat{\mu }$ and $\hat{\Sigma }$ by taking the derivative of μ and Σ and assigning these derivatives to be zeros. The estimated parameters are

(13) $$\hat{\mu }=\frac{1}{m}\sum_{i=1}^{m}D_{i},$$

(14) $$\hat{\Sigma }=\frac{1}{m}\sum_{i=1}^{m}\left(D_{i}-\hat{\mu }\right){\left(D_{i}-\hat{\mu }\right)}^{T}.$$

To obtain a good estimate for the covariance matrix, generally, we need to collect sufficient samples. For example, in this study, if we use a sequence of samples whose size is smaller than or equal to the dimension of the multivariate Gaussian distribution, it will be much likely that the estimated covariance matrix is extremely deviant from the ground truth or even singular. Furthermore, if there is perfect collinearity in the data, the covariance matrix will also be non-invertible. We adopt a regularization approach to tackle this problem, which can be represented as

(15) $${\hat{\Sigma }}^{\prime }=\hat{\Sigma }+\gamma I,$$

where I is an identity matrix and γ>0 is a tunable scaling factor. In doing so, we can force the covariance matrix to be non-singular, which makes the proposed approach applicable to the cases where the samples for the estimation are insufficient (more generally, to the cases where the covariance matrix is non-invertible).

### 5.3. Offline Fingerprint Generation Module

The offline fingerprint generation module aims to construct the radio map to store the information of the reference locations. Specifically, for a reference location T, the CSI amplitudes or phases collected from the link $l_{i}$ will be first input to the processing module to estimate the mean vector and covariance matrix, and then these parameters will be stored in the radio map as the fingerprints. For example, when using the CSI amplitudes or phases to construct the fingerprints, if there are a total of *L_α_* links, we can represent the fingerprint of the location T as

(16) $$F_{T}=\left\{\left({\hat{v}}_{1},{\hat{\Sigma }}_{1}\right),\ldots ,\left({\hat{v}}_{L_{\alpha }},{\hat{\Sigma }}_{L_{\alpha }}\right)\right\}.$$

We note that $F_{T}$ actually represents a set of multivariate Gaussian distributions, and therefore, we further write Equation (16) as

(17) $$F_{T}=\left\{f_{1},\ldots ,f_{L_{\alpha }}\right\},$$

where $f_{i}$ is the multivariate Gaussian distribution of the i-th communication link.

Moreover, in this study, we propose a kind of combined fingerprints, which incorporate the amplitude fingerprints and the phase fingerprints into a whole. The combined fingerprints make it possible to localize a target by simultaneously using both the amplitude information and the phase information. Specifically, for a reference location T, we define its amplitude fingerprint as $F_{T}^{a}$ and its phase fingerprint as $F_{T}^{p}$. Then, the combined fingerprint is

(18) $$F_{T}^{c}=\left\{F_{T}^{a},F_{T}^{p}\right\}.$$

We hold an assumption that $F_{T}^{a}$ and $F_{T}^{p}$ are independent of one another, thus simplifying the online KL-divergence based localization process which is presented in the below part.

### 5.4. Online KL-Divergence Based Localization Module

The online phase aims to estimate the target’s location using the testing fingerprints formed by the processing module. In this study, we adopt a function to compute the probabilities that a testing fingerprint belongs to all the fingerprints in the radio map. To estimate the location information of a target, we use these estimated probabilities as weights to average the reference locations. The details are illustrated below.

#### 5.4.1. KL-Divergence Based Kernel Function

The KL-divergence is a measure used to calculate the ‘distance’ (or ‘dissimilarity’) between two distributions [37], and the KL-divergence between two density p and q can be written as

(19) $$KL\left(p||q\right)=\int p\log\frac{p}{q}.$$

KL(p||q) is non-negative and equals zeros only if p=q. However, Equation (19) is non-symmetric, meaning that KL(p||q) is not equal to KL(q||p). Therefore, we introduce the symmetrized KL-divergence $D_{s}$, written as

(20) $$D_{s}\left(p,q\right)=KL(p||q)+KL(q||p).$$

Furthermore, the KL-divergence between two multivariate Gaussian distributions *f* and g is defined as

(21) $$KL(f||g)=\frac{1}{2}\left(tr\left(\Sigma_{g}^{-1}\Sigma_{f}\right)+{\left(\mu_{g}-\mu_{f}\right)}^{T}\Sigma_{g}^{-1}\left(\mu_{g}-\mu_{f}\right)-log\left(\frac{det\left(\Sigma_{f}\right)}{det\left(\Sigma_{g}\right)}\right)-\xi \right).$$

where ξ is the dimension of the multivariate Gaussian distribution. By combining Equations (20) and (21), we can derive the symmetrized KL-divergence $D_{s}$ between two arbitrary multivariate Gaussian distributions. Moreover, because of the assumption that different communication links are independent of each other, the symmetrized KL-divergence of two arbitrary fingerprints $F^{\upsilon }$ and $F^{\tau }$ can be represented as follows according to the chain rule of KL-divergence [38].

(22) $$D_{s}\left(F^{\upsilon },F^{\tau }\right)=\overset{L_{\alpha }}{\underset{i=1}{\sum }}D_{s}\left(f_{i}^{\upsilon },f_{i}^{\tau }\right).$$

According to [37], a kernel function can be defined as follows to transform the symmetrized KL-divergence to a measure whose value is between 0 and 1

(23) $$S_{f}\left(F^{\upsilon },F^{\tau }\right)=e^{-\alpha D_{s}\left(F^{\upsilon },F^{\tau }\right)},$$

where α>0 is the scaling factor whose value is dependent on the data. It is easy to see that if and only if $F^{\upsilon }$ is equal to $F^{\tau }$, the function will output 1. Otherwise, the function will output a value between 0 and 1. Furthermore, we can consider that Equation (23) presents a metric indicating how similar $F^{\upsilon }$ and $F^{\tau }$ are, and by incorporating α, we can obtain a much more flexible and controllable measure.

#### 5.4.2. Localization with the CSI Amplitude or Phase

In this part, we introduce the process of localizing a target using merely the CSI amplitudes or phases, and the localization process by combining both of them is presented in the next part.

To estimate a target’s location, we adopt a probabilistic approach, written as

(24) $$\mathrm{Pr}\left(T_{i}|F\right)=\frac{\mathrm{Pr}\left(F,T_{i}\right)}{\mathrm{Pr}\left(F\right)}=\frac{\mathrm{Pr}\left(T_{i}\right)\mathrm{Pr}\left(F|T_{i}\right)}{\sum_{i=1}^{N_{L}}\mathrm{Pr}\left(T_{i}\right)\mathrm{Pr}\left(F|T_{i}\right)},$$

where $T_{i}$ is the i-th reference location, *F* is the testing fingerprint, $\mathrm{Pr}\left(T_{i}|F\right)$ denotes the posterior probability of $T_{i}$, $\mathrm{Pr}\left(T_{i}\right)$ represents the priori probability, and $N_{L}$ is the number of reference locations. Then, we assume that there is no bias among different reference locations, meaning that $\mathrm{Pr}\left(T_{i}\right)$ is equal to 1/*N_L_*. Therefore, Equation (24) can be simplified as

(25) $$\mathrm{Pr}\left(T_{i}|F\right)=\frac{\mathrm{Pr}\left(F|T_{i}\right)}{\sum_{i=1}^{N_{L}}\mathrm{Pr}\left(F|T_{i}\right)}.$$

We utilize Equation (23) to calculate $\mathrm{Pr}\left(F|T_{i}\right)$, written as

(26) $$\mathrm{Pr}\left(F|T_{i}\right)=e^{-\alpha D_{s}\left(F,F_{T_{i}}\right)}.$$

Finally, the estimated location is

(27) $$\hat{T}=\overset{N_{L}}{\underset{i=1}{\sum }}\mathrm{Pr}\left(T_{i}|F\right)T_{i}.$$

#### 5.4.3. Localization with the Combination of the CSI Amplitude and Phase

According to the combined fingerprints presented above in Equation (18), we modify Equation (25) as follows so that we can perform localization simultaneously using the CSI amplitude and phase information

(28) $$\mathrm{Pr}\left(T_{i}|F^{c}\right)=\frac{\mathrm{Pr}\left(F^{a},F^{p}|T_{i}\right)}{\sum_{i=1}^{N_{L}}\mathrm{Pr}\left(F^{a},F^{p}|T_{i}\right)}.$$

Further, as we have assumed that $F^{a}$ and $F^{p}$ are independent of one another, therefore, we have

(29) $$\mathrm{Pr}\left(F^{a},F^{p}|T_{i}\right)=\mathrm{Pr}\left(F^{a}|T_{i}\right)\cdot \mathrm{Pr}\left(F^{p}|T_{i}\right)=e^{-\alpha^{a}D_{s}\left(F^{a},F_{T_{i}}\right)-\alpha^{p}D_{s}\left(F^{p},F_{T_{i}}\right)},$$

Then, by substituting Equation (29) into Equation (28), we can obtain the value of $\mathrm{Pr}\left(T_{i}|F^{c}\right)$. Finally, according to Equation (27), we can obtain the estimated location.

## 6. Evaluation

### 6.1. Experimental Details

We implemented the proposed approach in two typical indoor environments to test its efficiency. In both scenarios, we adopt a scheme of one Access Point (AP) and one Monitor Point (MP). The AP is a TP-Link router, and the MP is an *HP* laptop installed with an intel 5300 wireless card. To collect the raw CSI measurements, we installed CSITOOL on the laptop. In this study, we collect 100 consecutive samples at each location to construct the fingerprints. For the proposed approach, we chose to use two communication links out of three to perform the localization task. For Pilot, only one antenna was selected. For Nuzzer, in the corridor testbed, we chose to use one communication link, and in the laboratory testbed, two communication links were selected. Furthermore, for a fair comparison, we also performed the weighted averaging, the same as the proposed approach, in Pilot, and when implementing Nuzzer, we used its continuous space estimator to average the reference locations.

**Figure 12:**
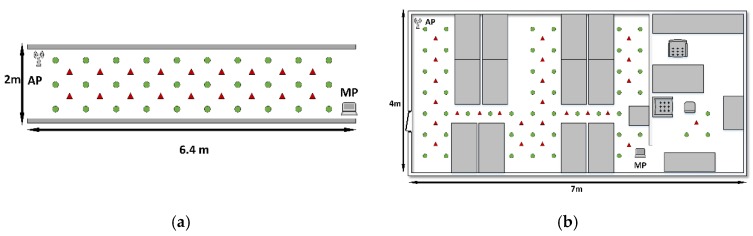
Layouts of (**a**) the corridor and (**b**) laboratory.

We show the layout of the two scenarios in the Figure 12, and the details of them are as follows:

1. Corridor: the corridor environment has a size of 2 m×6.4 m, which has no obstacle in its area. However, the space of the monitoring area is fairly narrow, which may increase the effect of multipath. As is shown in Figure 12a, there are a total of 30 reference locations and 18 testing locations uniformly distributed in the monitoring area.
2. Laboratory: as shown in Figure 12b, the laboratory is composed of two rooms, which are divided by a screen. The size of the large one is about 4 m×5 m, whereas the small one has an area of around 4 m×2 m. This scenario is overwhelmed by extremely strong multipath effects and interventional signals, which may render the CSI measurements unstable.

The detailed configuration of the two scenarios are listed in Table 2. The performance metric used in this paper is the mean distance error, which is

(30) $$err=\frac{1}{C}\overset{C}{\underset{i=1}{\sum }}\sqrt{{\left({\hat{x}}_{i}-x_{i}\right)}^{2}+{\left({\hat{y}}_{i}-y_{i}\right)}^{2}}.$$

where C is the total number of the testing locations, $\left({\hat{x}}_{i},{\hat{y}}_{i}\right)$ is the location estimate, and $\left(x_{i},y_{i}\right)$ is the ground truth.

### 6.2. Localization Performance

To test the performance of the proposed approach, we compared it with two different state-of-the-art systems, namely Pilot and Nuzzer. Also, we tested the proposed approach with different types of fingerprints.

The results of our experiments are listed in Table 3. In the corridor environment, when adopting the combined fingerprints, the mean distance error of the proposed approach is 0.94665 m by using two communication links, and $\alpha^{a}$ and $\alpha^{p}$ are set to be 9 × 10^−4^ and 3 × 10^−2^. When merely using the CSI amplitude, with α set to be 3 × 10^−3^, we obtain a worse result, which is 0.99716 m. For the situation where we only use the phase information, we obtain a localization error of 1.04339 m by setting α to be 3 × 10^−2^. Pilot, in this case, achieves a localization error of 1.24999 m, whereas the proposed approach, whatever type of fingerprints is used, outperforms Pilot. Nuzzer, which exploits RSSI to perform localization, has a localization error of merely 1.46679 m, worse than the proposed approach and Pilot.

In the laboratory testbed, which is cluttered with office appliances, the multipath effect is very strong, making localization accuracy degraded. The proposed approach has a localization error of 1.34747 m when using the combined fingerprints, with $\alpha^{a}$ and $\alpha^{p}$ set to be 4 × 10^−3^ and 6 × 10^−5^. In comparison, when using merely the CSI amplitudes, the proposed approach has slightly worse performance, which is 1.35196 m with α set to be 4 × 10^−3^. When only using the phase fingerprints, we set α to be 5 × 10^−3^, finally achieving a localization error of 1.55726 m. The other two approaches, in this case, have poor performance, with Pilot to be 1.74823 m and Nuzzer 1.80899 m, both worse than the proposed approach.

**Figure 13:**
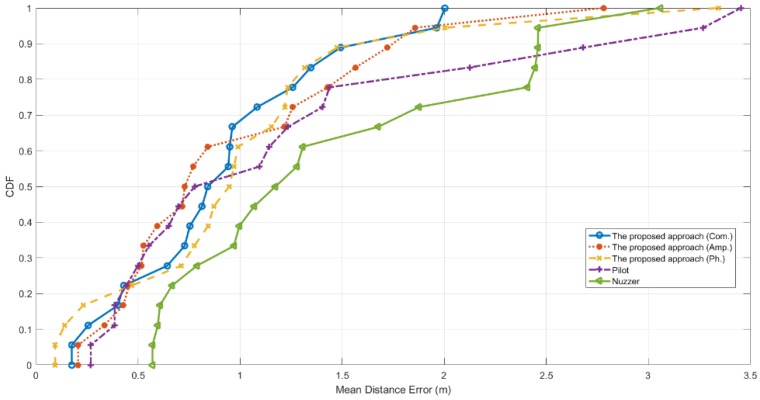
Cumulative Distribution Function (CDF) of the mean distance error tested in the corridor testbed.

Figure 13 shows the Cumulative Distribution Function (CDF) of the distance error in the corridor scenario. In this testbed, Pilot and the proposed approach with the amplitude fingerprints make sure that 50% of the test locations have a localization error under 0.72 m. When using the CSI phase information, the proposed approach has 50% of the test locations under 0.9 m, and that value achieved by exploiting the combined fingerprints is 0.82 m. Nuzzer, in this case, merely achieves a result of 50% under 1.1 m. Furthermore, the proposed approach, with the phase fingerprints or the combined fingerprints, accomplishes that 80% of the test locations are well below 1.3 m. For the proposed approach using the amplitude information, 80% of the test locations have a localization accuracy of merely below 1.45 m, so does Pilot. Nuzzer has 80% of the test locations below 2.45 m, performing worse than the other CSI-based approaches.

**Figure 14:**
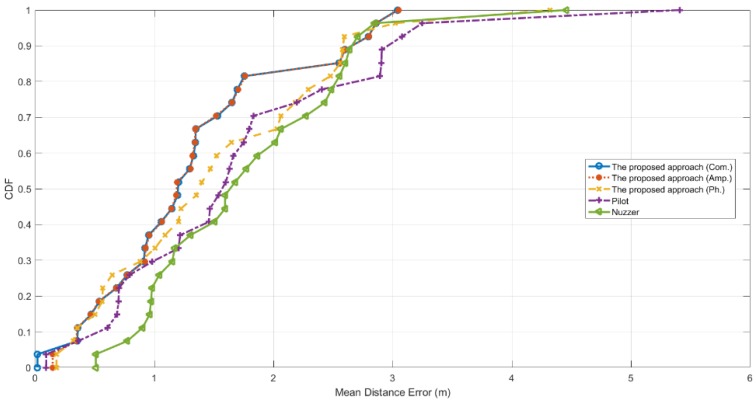
CDF of the mean distance error tested in the laboratory testbed.

Figure 14 shows the CDF results tested in the laboratory room. In this case, we will not bother to describe much the results of the proposed approach with the combined fingerprints, because it merely achieves better performance at one testing location compared to that of using the amplitude fingerprints. According to Figure 14, the two curves almost overlap except an apparent difference at a testing location at the lower-left part of the figure. We can see that at that location, by employing the combined fingerprints we achieve a localization error of 0.0193 m, whereas it is 0.14725 m by merely using the amplitude fingerprints.

In this testbed, though the environment is cluttered, the proposed approach still achieves rather good performance that the localization errors of about 50% of the testing locations are below 1.20 m with the amplitude fingerprints, and 1.35 m with the phase fingerprints. Pilot achieves that 50% of the test locations are under 1.55 m. Nuzzer has 50% of the testing locations merely under 1.6 m. Furthermore, for the proposed approach, the localization errors of 80% of the testing locations are well below 1.75 m and 2.3 m with respectively the amplitude fingerprints and the phase fingerprints, whereas those of Pilot and Nuzzer are about 2.45 m and 2.50 m respectively.

According to the results, we can see that the proposed approach, which utilizes multiple communication links and the uncertainty information of CSI, performs better than Pilot in both testbeds, no matter what type of fingerprints is used. The better results of the proposed approach and Pilot than that of Nuzzer demonstrate the advantage of CSI that multiple subcarriers provide more useful information. In comparison, RSSI has merely one integer value, merely providing rather coarse information about the quality of a communication link.

### 6.3. Influence of the Parameters

In this section, we explore the effects of the parameter selections on the localization accuracy, including the combination of communication links, selection of the type of fingerprints, number of packets, and value of the scaling factor.

#### 6.3.1. Combination of Communication Link

To study the influence of different communication link combinations on the localization accuracy, we experimented several times in each environment. We denote the three links as a, b, and c respectively. Further, a–b represents the combination of a and b, a–b–c represents the combination of a, b and c, and so on.

**Figure 15:**
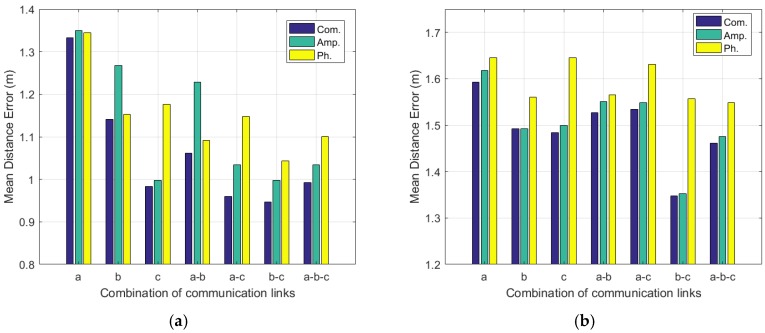
Mean distance errors under different situations in (**a**) the corridor and (**b**) laboratory.

As we can see from Figure 15a, in the corridor scenario, when using b–c, we obtain the best accuracy among all the link combinations, no matter what type of fingerprints is used. Moreover, using a–b have better performance than that of using merely a or b, whatever type of fingerprints is used. Utilizing a–c, we can achieve better performance than that of using merely a or *c* under the condition of adopting the combined fingerprints or the phase fingerprints. Furthermore, by combining all the three links, we can obtain lower localization errors than the situations of adopting merely a or b, no matter what type of fingerprints is used. However, a–b–c cannot beat the single link *c* when employing the amplitude fingerprints or the combined fingerprints.

The results of the laboratory are shown in Figure 15b. We can see that the best performance is achieved when employing b–c, for whatever type of fingerprints. By adopting *a*–*b*–*c*, we can obtain the suboptimal results, for an arbitrary type of fingerprints. However, using a–b do not yield lower localization errors than using b, but it still produces better results than the single link a. We can see a similar result when using a–c, where it achieves lower localization errors than the single link a but higher localization errors than the link c, on the condition of adopting the amplitude fingerprints or the combined fingerprints. Meanwhile, with the phase fingerprints, a–c beats the single link a and *c*.

According to the results, we notice that combining multiple communication links is a reasonable way of enhancing localization accuracy, but it does not necessarily produce an improved result. Therefore, a careful selection of the communication link combination is needed.

#### 6.3.2. Selection of the Type of Fingerprints

In this part, we explore the effects of using different types of fingerprints, namely the amplitude fingerprints, phase fingerprints, and combined fingerprints. The results are shown in Figure 15. We can see that, in the corridor room, simultaneously using the combined fingerprints has the best performance among all the communication link combinations. Additionally, employing the amplitude fingerprints is much more likely to achieve better performance than the phase fingerprints.

In the laboratory scenario, we observe similar results to those in the corridor. In the cases of except b, adopting the combined fingerprints can obtain a bit lower localization errors than merely using the amplitude fingerprints or the phase fingerprints. However, we notice that combining amplitude and phase do not necessarily improve performance, which is also shown in the results of the case b. In the case b, the phase information has no positive contribution to the localization accuracy improvement but negative effects. By setting $\alpha^{p}$ to be nearly 0, we can approximately eliminate the effects of the phase information, thus making the localization accuracy nearly equivalent to that of the amplitude fingerprints. Moreover, in this case, it is pointless to generate the combined fingerprints to localize a target, because no accuracy improvement will be seen, and if the parameters are not carefully selected, we may obtain a degraded result. For example, when we set $\alpha^{a}$ and $\alpha^{p}$ to be 0.01, we will obtain a localization error of 1.52433 m with the amplitude fingerprints, and 1.56442 m with the phase fingerprints. However, by utilizing the combined fingerprints, the localization error is 1.59327 m, worse than the other two situations.

Another observation is that in the laboratory, the results of the amplitude fingerprints are better than those of the phase fingerprints in all the communication link combinations.

According to our results, we conclude that it is hard to tell which is better, amplitude or phase, but usually, utilizing the amplitude fingerprints is more likely to yield a better result than using the phase fingerprints. Furthermore, combining the CSI amplitude and phase, generally, achieves better results than merely using either of them, but there may also be some cases where the combination of amplitude and phase has no localization accuracy improvement.

#### 6.3.3. Number of Packets (η)

To obtain the mean vectors and covariance matrices used for the construction of the fingerprints, we need to collect enough packets, thus yielding accurate estimates. In this part, we conducted several experiments to explore the effects of this parameter. Specifically, for situations where the packets number η is smaller than or equal to 30, we enforce regularization to make sure we can obtain a relatively good estimate, where the regularization term is set to be 1 × 10^−10^.

The results are shown in Table 4. In the corridor testbed, when η is smaller than or equal to 30, we observe rather bad performance whatever type of fingerprints is used. On the whole, with the increase of the value of η, the mean distance error displays a decreasing trend, except a spike when η equals 30. The localization performance then starts to become roughly stable after η is equal to 50.

In the laboratory room, the localization error of the proposed approach with the phase fingerprints keeps rather stable when *η* is smaller than or equal to 50, and with η reaching 100, it plummets to about 1.55 m and then keeps stable. This may imply that 100 consecutive packets are enough for a good estimate in this testbed when the phase fingerprints are used, whereas 50 packets are not.

Again, we will not spare too much effort to discuss the results of the combined fingerprints for the reason mentioned in Section 6.2 and in the rest of this paragraph; all the focus will be put on the situation where the amplitude fingerprints are used. We can see that the results of the amplitude fingerprints are oscillating before η reaches 50 and become stable after that value. We consider that this phenomenon also reflects that in this testbed, a small number of packets, say less than or equal to 40, is not sufficient to obtain stable performance.

According to our results, we observe that the localization error is sensitive to the value of η. When the value is too small, it may be possible that we cannot produce a good result. With the increase of its value, the results are likely to become stable, but there might be fluctuations over accuracy. In this study, we think using 100 consecutive packets is a nice choice. The reason why we choose 100 is that we hope to obtain a sufficiently good estimate for the mean vectors and covariance matrices without too much time delay or device burden. For the cases where η is less than 100, the results are likely to be unstable. When it is greater than 100, we either need more time to collect the samples or have to increase the sampling rate, which will impose more burden on the devices. Our device can transmit packets at a rate of 100 or 200 per second easily, and the time needed for collecting samples is less than or equal to 1 s, which is reasonable. Therefore, we think choosing 100 consecutive samples is a good tradeoff.

#### 6.3.4. The Value of the Scaling Factor (γ)

In this part, we explore the effects of the scaling factor. As we have articulated, when the size of the sequence is insufficient for a good estimate, it will make the estimated covariance matrix deviant from the ground truth or even possibly non-invertible. In these cases, the regularization is needed to force the covariance matrix to exhibit non-singularity. In our study, when the size of the sequence is smaller than or equal to 30, it is likely that we will obtain a bad estimate of the covariance matrix. We use the results when *η* is 10 to display the effects of different values of *γ* on localization performance, as in Table 5.

In the corridor testbed, the proposed approach with the amplitude fingerprints or the combined fingerprints has fairly stable performance with the increase of the value of *γ*. For the situation of using the phase fingerprints, with the increase of the value of *γ*, the localization error first keeps stable and then plummet to about 1.19 m when γ reaches 1 × 10^0^.

In the laboratory room, the proposed approach with the amplitude fingerprints or combined fingerprints exhibit a gradually increasing trend. At first, the localization error keeps stable, but starts to rise when γ reaches 1 × 10^−2^ and keeps surging. The proposed approach with the phase fingerprints displays a downward trend, whose localization error also keeps stable at first but begins to decrease slightly with γ reaching 1 × 10^−4^.

Also, we tested the situations without regularization. We have to say that although in these cases we obtained the inverse of the covariance matrices, one thing for sure is that the inverse is considerably deviant from the ground truth. According to Table 6, we can see that the results without regularization are rather bad compared to those with regularization.

Our results show that in different scenarios, the sensitivity of the value of γ to the localization error is different, and different types of fingerprints have diverse sensitivity to this parameter. Furthermore, we note that adding a regularization term with a small value is sufficient for improving the localization performance in such ill-conditioned situations.

## 7. Conclusions

In this paper, we propose a novel approach, which utilizes the uncertainty of CSI, embodied by the probability distribution, to implementing target localization in a device-free manner. Firstly, we show that the Gaussian distribution can be used to model the CSI sanitized phases of a subcarrier. Furthermore, we also model the CSI amplitudes of a subcarrier as a Gaussian distribution. Then, we show that the mean vectors and covariance matrices of the CSI amplitudes or sanitized phases may display different patterns when a target stands at different locations. Therefore, we model the CSI amplitudes or the sanitized phases of the subcarriers within a communication link as a multivariate Gaussian distribution to further exploit these differences. Further, we use multiple communication links to boost useful information. To localize the target, we utilize the symmetrized KL-divergence to calculate the ‘dissimilarity’ of a testing fingerprint with the fingerprints in the radio map. Next, we adopt a kernel function to transform the ‘dissimilarity’ to the form of probability. By considering the probabilities as the weights, we can obtain the location estimate with a weighted averaging method. Moreover, the proposed approach can process three types of fingerprints, namely the amplitude fingerprints, phase fingerprints, and combined fingerprints.

We conduct extensive experiments to demonstrate the effectiveness of the proposed approach and also explore the effects of the choices of different parameters on the localization error. The experimental results show that the proposed approach achieves good performance in two typical indoor environments.

In this study, we do not take location tracking into consideration, which may be part of our future work. The incorporation of the fingerprinting-based approaches and the model-based ones may also be part of our future work. Furthermore, we merely assume that the different communication links are independent of one another and that the CSI amplitude and sanitized phase of a subcarrier are also independent of each other, and the study of their relationships may be part of our future work.

## Figures and Tables

**Table 1:** Rejection ratios under different conditions.

Condition	Rejection Ratio
With no target	0.0556
With a target	0.1235

**Table 2:** Configurations of the two indoor scenarios.

Testbed	Parameter	Value
Corridor	Antenna number of Access Point (AP)	1
	Antenna number of Monitor Point (MP)	3
	Number of reference locations	30
	Number of testing locations	18
Laboratory	Antenna number of AP	1
	Antenna number of MP	3
	Number of reference locations	50
	Number of testing locations	27

**Table 3:** Localization performance in two typical indoor scenarios.

Testbed	Approach	Mean Distance Error (m)
Corridor	The proposed approach (Com. ^1^)	0.94665
	The proposed approach (Amp.^2^)	0.99716
	The proposed approach (Ph. ^3^)	1.04339
	Pilot	1.24999
	Nuzzer	1.46679
Laboratory	The proposed approach (Com.)	1.34747
	The proposed approach (Amp.)	1.35196
	The proposed approach (Ph.)	1.55726
	Pilot	1.74823
	Nuzzer	1.80899

^1^ Com. indicates the situation where the combined fingerprints are used; ^2^ Amp. indicates the situation where the amplitude fingerprints are used; ^3^ Ph. indicates the situation where the phase fingerprints are used; we maintained the usage in the rest of the figures and tables for simplicity.

**Table 4:** Mean distance errors using different number of packets.

Testbed	Type of Fingerprints	Mean Distance Errors (m) of Different Number of Packets						
		10	20	30	40	50	100	200
Corridor	Amp.	1.08779	1.06805	1.26485	0.99898	1.00424	0.99716	0.99873
	Ph.	1.29895	1.26187	1.38441	1.13489	1.10677	1.04339	1.10052
	Com.	1.07382	1.06805	1.10785	0.99277	0.96604	0.94665	0.98174
Laboratory	Amp.	1.38741	1.32088	1.49950	1.40812	1.35618	1.35196	1.36101
	Ph.	1.60903	1.59592	1.65660	1.59859	1.60923	1.55726	1.55806
	Com.	1.38550	1.32029	1.49950	1.40810	1.35618	1.34747	1.36101

**Table 5:** The effects of different values of γ on mean distance error.

Testbed	Type of Fingerprints	Mean Distance Errors (m) of Different Values of γ						
		1 × 10^−10^	1 × 10^−8^	1 × 10^−6^	1 × 10^−4^	1 × 10^−2^	1 × 10^0^	1 × 10^2^
Corridor	Amp.	1.08779	1.08779	1.08779	1.08778	1.08678	1.06790	1.09679
	Ph.	1.29894	1.29895	1.29903	1.30536	1.23779	1.18977	1.18766
	Com.	1.07382	1.07382	1.07386	1.07604	1.03481	1.05284	1.07542
Laboratory	Amp.	1.38741	1.38741	1.38741	1.38746	1.39048	1.47399	1.51659
	Ph.	1.60903	1.60903	1.60886	1.59939	1.58294	1.58142	1.58137
	Com.	1.38550	1.38550	1.38550	1.38520	1.38748	1.47224	1.51659

**Table 6:** Mean distance error without regularization.

Testbed	Type of Fingerprints	Mean Distance Error (m)
Corridor	Amp.	1.25487
	Ph.	1.36112
	Com.	1.25195
Laboratory	Amp.	1.65794
	Ph.	1.65703
	Com.	1.65703

## References

[1] Youssef M., Mah M., Agrawala A. “Challenges: Device-free Passive Localization for Wireless”. Proceedings of the ACM International Conference on Mobile Computing and Networking (MobiCom).

[2] Seifieldin M., Saeed A., Kosba A., Keyi A., Youssef M. (2013). Nuzzer: A Large-Scale Device-Free Passive Localization System for Wireless Environments. IEEE Trans. Mob. Comput..

[3] Xu C., Firner B., Zhang Y., Howard R., Li J., Lin X. Improving RF-Based Device-Free Passive Localization in Cluttered Indoor Environments Through Probabilistic Classification Methods. Proceedings of the 11th International Conference on Information Processing in Sensor Networks.

[4] Halperin D., Hu W., Sheth A., Wetherall D. (2011). Tool Release: Gathering 802.11n Traces with Channel State Information. ACM SIGCOMM Comput. Commun. Rev..

[5] Xiao J., Wu K., Yi Y., Wang L., Ni L.M. Pilot: Passive device-free indoor localization using channel state information. Proceedings of the 33rd International Conference on Distributed Computing Systems (ICDCS).

[6] Zhou R., Xiang L., Zhao P., Chen J. (2017). Device-Free Presence Detection and Localization with SVM and CSI Fingerprinting. IEEE Sens. J..

[7] Want R., Hopper A., Falcao V., Gibbons J. (1992). The active badge location system. ACM Trans. Inf. Syst..

[8] Aparicio S., Perez J., Tarrio P., Bernardos A.M., Casar J.R. An Indoor Location Method Based on a Fusion Map Using Bluetooth and WLAN Technologies. Proceedings of the International Symposium on Distributed Computing and Artificial Intelligence.

[9] Ni L.M., Liu Y., Lau Y.C., Patil A.P. (2004). LANDMARC: Indoor Location Sensing Using Active RFID. Wirel. Netw..

[10] Hazas M., Hopper A. (2006). Broadband ultrasonic location systems for improved indoor positioning. IEEE Trans. Mobile Comput..

[11] Bahl P., Padmanabhan V.N. Radar: An in-building RF-based user location and tracking system. Proceedings of the IEEE INFOCOM 2000. Conference on Computer Communications. Nineteenth Annual Joint Conference of the IEEE Computer and Communications Societies.

[12] Youssef M., Agrawala A. (2008). The Horus Location Determination System. Wirel. Netw..

[13] Tian Z.S., Li Z., Zhou M., Jin Y., Wu Z.P. (2016). PILA: Sub-meter localization using CSI from commodity Wi-Fi devices. Sensors.

[14] Wu K., Xiao J., Yi Y., Gao M., Ni L.M. FILA: Fine-grained indoor localization. Proceedings of the IEEE INFOCOM.

[15] Xiao J., Wu K.S., Yi Y.W., Ni L.M. FIFS: Fine-Grained Indoor Fingerprinting System. Proceedings of the 2012 21st International Conference on Computer Communications and Networks.

[16] Chapre Y., Ignjatovic A., Seneviratne A., Jha S. (2015). CSI-MIMO: An efficient Wi-Fi fingerprinting using Channel State Information with MIMO. Pervasive Mob. Comput..

[17] Zheng L.L., Hu B.J., Chen H.X. (2018). A high accuracy time-reversal based wifi indoor localization approach with a single antenna. Sensors.

[18] Wang Y., Xiu C., Zhang X., Yang D. (2018). WiFi Indoor Localization with CSI Fingerprinting-Based Random Forest. Sensors.

[19] Wang X., Gao L., Mao S., Pandey S. (2016). CSI-based fingerprinting for indoor localization: A deep learning approach. IEEE Trans. Veh. Technol..

[20] Wang X., Gao L., Mao S. (2016). CSI Phase Fingerprinting for Indoor Localization with a Deep Learning Approach. IEEE Internet Things J..

[21] Chen H., Zhang Y., Li W., Tao X., Zhang P. (2017). ConFi: Convolutional Neural Networks Based Indoor Wi-Fi Localization Using Channel State Information. IEEE Access.

[22] Wilson J., Patwari N. (2010). Radio Tomographic Imaging with Wireless Networks. IEEE Trans. Mob. Comput..

[23] Wilson J., Patwari N. (2011). See through walls: Motion tracking using variance-based radio tomography networks. IEEE Trans. Mob. Comput..

[24] Zhao Y., Patwari N. (2015). Robust estimators for variance-based device-free localization and tracking. IEEE Trans. Mob. Comput..

[25] Adib F., Kabelac Z., Katabi D., Miller R.C. 3D Tracking via Body Radio Reflections. Proceedings of the 11th USENIX Conference on Networked Systems Design and Implementation.

[26] Li X., Li S., Zhang D., Xiong J., Wang Y., Mei H. (2016). Dynamic-music: Accurate device-free indoor localization. Proceedings of the 2016 ACM International Joint Conference on Pervasive and Ubiquitous Computing.

[27] Li X., Zhang D., Lv Q., Xiong J., Li S., Zhang Y., Mei H. (2017). IndoTrack: Device-Free Indoor Human Tracking with Commodity Wi-Fi. Proc. ACM Interact. Mob. Wearable Ubiquitous Technol..

[28] Qian K., Wu C., Yang Z., Jamieson K. Widar: Decimeter-Level Passive Tracking via Velocity Monitoring with Commodity Wi-Fi. Proceedings of the 18th ACM International Symposium on Mobile Ad Hoc Networking and Computing.

[29] Qian K., Wu C., Zhang Y., Zhang G., Yang Z., Liu Y. Widar2.0: Passive Human Tracking with a Single Wi-Fi Link. Proceedings of the 16th Annual International Conference on Mobile Systems, Applications, and Services.

[30] Xiao W., Song B., Yu X., Chen P. (2015). Nonlinear Optimization-Based Device-Free Localization with Outlier Link Rejection. Sensors.

[31] Zhang J., Xiao W., Zhang S., Huang S.D. (2017). Device-free Localization via an Extreme Learning Machine with Parameterized Geometrical Feature Extraction. Sensors.

[32] Zhang J., Xiao W., Li Y., Zhang S. (2018). Residual compensation extreme learning machine for regression. Neurocomputing.

[33] Zhang J., Xiao W., Li Y., Zhang S., Zhang Z. Multilayer probability extreme learning machine for device-free localization. Neurocomputing.

[34] Gao R., Xue J., Xiao W., Zhao B., Zhang S. Extreme Learning Machine Ensemble for CSI based Device-free Indoor Localization. Proceedings of the 2019 28th Wireless and Optical Communications Conference (WOCC).

[35] Gao Q., Wang J., Ma X., Feng X., Wang H. (2017). CSI-Based Device-Free Wireless Localization and Activity Recognition Using Radio Image Features. IEEE Trans. Veh. Technol..

[36] Qian K., Wu C., Yang Z., Liu Y., Zhou Z. PADS: Passive Detection of Moving Targets with Dynamic Speed using PHY Layer Information. Proceedings of the 20th IEEE International Conference on Parallel and Distributed Systems (ICPADS).

[37] Moreno P., Ho P., Vasconcelos N. A kullback-leibler divergence based kernel for SVM classification in multimedia applications. Proceedings of the 17th Annual Conference on Neural Information Processing Systems (NIPS).

[38] Cover T.M., Thomas J.A. (2006). Entropy, relative entropy, and mutual information. Elements of Information Theory.

